# Environmental exposure to arsenic may reduce human semen quality: associations derived from a Chinese cross-sectional study

**DOI:** 10.1186/1476-069X-11-46

**Published:** 2012-07-09

**Authors:** Weipan Xu, Huaqiong Bao, Feng Liu, Liangpo Liu, Yong-Guan Zhu, Jianwen She, Sijun Dong, Min Cai, Lianbing Li, Chuanhai Li, Heqing Shen

**Affiliations:** 1 Key Lab of Urban Environment and Health, Institute of Urban Environment, Chinese Academy of Sciences, 1799 Jimei Road, Xiamen 361021, China; 2 Chongqing Key Laboratory of Birth Defects and Reproductive Health, The Affiliated Hospital of Chongqing Institute for Population and Family Planning, Chongqing 400020, China; 3 Environmental Health Laboratory Branch, California Department of Public Health, Richmond, CA 94804, USA

**Keywords:** Arsenic, China, Cross-sectional study, Human reproduction, Semen quality

## Abstract

**Background:**

Recent observations in *in vitro* and *in vivo* models suggest that arsenic (As) is an endocrine disruptor at environmentally-relevant levels. When exposed to As, male rats and mice show steroidogenic dysfunction that can lead to infertility. However, the possible effects of As on human male semen quality remain obscure.

**Methods:**

We monitored the profile of As species in the urine of a reproductive-age human cohort and assessed its association with semen quality. Men (*n* = 96) were recruited in an infertility clinic from July 2009 to August 2010 in the Affiliated Hospital of Chongqing Institute for Population and Family Planning. Five urinary As species were analyzed by high-performance liquid chromatography coupled with inductively coupled plasma mass spectrometry (HPLC-ICP-MS). Clinical information on the semen volume, sperm concentration and motility was employed to catalogue and evaluate semen quality according to WHO guidelines. As species concentrations in addition to other continuous variables were dichotomized by the medians and modelled as categorical variables in order to explore using the binary logistic regression possible associations between As exposure and semen quality.

**Results:**

Urinary concentrations (geometric mean ± SD, μg g^-1^ creatinine) of different As species were 7.49 (±24.8) for AsB, 20.9 (±13.7) for DMA, 2.77 (±3.33) for MMA, and 4.03 (±3.67) for As*i* (As*i*^III^ and As*i*^V^). DMA concentrations above the median were significantly associated with below-reference sperm concentrations (*P* =0.02) after adjusting for age, body mass index (BMI), abstinence, smoking and drinking habits. In addition, smoking was positively associated with MMA.

**Conclusion:**

Reduced parameters in human semen quality are positively associated with As exposure in a reproductive-age Chinese cohort.

## Background

Arsenic (As) is a widely-distributed element in nature, species of which are well-known toxicants. Exposure is associated with a range of adverse effects, including gastroenteritis, neurological manifestations, vascular changes, diabetes and cancers (bladder, lung, liver, kidney and prostate) [1]. It is possible to metabolize As into different species *via* several pathways; one is methylation, which is the major pathway for generation of inorganic As (As*i*) in the human body. It has been reported that the metabolic pathway for As*i* is a consequence of the following reaction: As*i*^V^ + 2e^–^ → As*i*^III^ + CH_3_^+^ → MMA^V^((CH_3_)AsO(OH)_2_) + 2e^–^ → MMA^III^ + CH_3_^+^ → DMA^V^((CH_3_)_2_AsOOH) + 2e^–^ → DMA^III^ + CH_3_^+^ → TMAO (trimethylarsine oxide) [2]. However, this reaction often does not fully metabolize As, and intermediaries, such as As*i*, MMA^III^ and MMA^V^, can remain in the body [3,4]. In geographical areas that do not have high levels of As contamination in drinking water, dietary intake is the major exposure route. Thus, consumption of contaminated foods or their processed products are often major contributors to As exposure and subsequent human-health effects.

Toxicity and bioavailability of As depend on its chemical speciation [5]. A number of mechanisms have been proposed to explain As’ influence on many disease processes, including possible alteration of cell signalling, cell cycle control, oxidative stress and DNA repair [6-9]. Recently, As has been recognized as an endocrine disruptor because it alters steroid and thyroid hormone receptor-mediated gene regulation at low and environmentally-relevant levels in cell culture and whole-animal models. The status of receptors for glucocorticoid, androgen, progesterone, mineralocorticoid, oestrogen, retinoic acid and thyroid hormones is altered [10-14].

As interference with oestrogen receptor (ER)-mediated gene expression [12,15-17] may disrupt the vast network of signalling pathways that are potentially influenced by oestrogens [18]. Although early studies [19,20] reported conflicting results regarding the As effects on fertility in male and female animals, more recent reports suggest that exposure alters female reproductive physiology [16]. Male rats and mice exposed to As exhibited steroidogenic dysfunction that possibly led to infertility [21-24]. It was suggested that chronic As exposure may contribute to male infertility in Comarca Lagunera, Mexico [25] and, has a negative impact on erectile function in a Taiwanese cohort [26]. However, few studies have determined the effects of As on semen quality. Based on the hypothesis that As exposure alters human spermatogenesis, As exposures in a reproductive-age cohort were determined by analyzing in urine various species (arsenobetaine (AsB), DMA^V^, MMA^v^, As*i*^III^ and As*i*^V^) and associating derived profiles with recommended parameters of semen quality.

## Materials and methods

### Participant recruitment and urine sample collection

The study was performed according to the Declaration of Helsinki, and the procedures were approved by the local ethics committee. Study participants were enrolled from July 2009 to August 2010 in the Affiliated Hospital of Chongqing Institute for Population and Family Planning, Chongqing, China. All male partners in infertile couples were asked to participate, without additional exclusion criteria. Every volunteer participant was informed about the purpose of this research, and written informed consent obtained. All participants were married but were childless for unknown reasons. The length of the respective couples’ infertility was not recorded. Cross-sectional questionnaires were employed in meeting sessions conducted by trained interviewers who recorded general participant characteristics, including demographics (age, height, weight, religion, etc.), smoking and drinking habits, education status and medical histories. Participants without smoking or drinking histories in the last year were defined as non-smokers or non-drinkers. Male semen quality was assessed and associations with As exposure were explored.

All study participants contributed at least one urine sample. 55% of the participants contributed two urine samples on separate occasions. For those who contributed two samples, the entire first urine sample was collected on the same day as the semen sample; the mean number of subsequent days to collection of the second sample was 12. 77% of these study participants contributed their second urine sample within 1–7 days from the first contribution. Among the other study participants who contributed two urine samples, 7.3% of the second urine samples were collected within 12–50 days, and 5.2% were collected within 66–180 days post-contribution of the initial samples. The half-life of inorganic arsenic is only 1.5(±0.9) day in the human body [27]. For those contributing two samples, the mean of both derived measurements was used in subsequent analysis. The samples were initially stored at −40°C, transported to the analytical laboratory in Xiamen packed in dry ice, and then kept at −80°C until analysis.

### Semen collection and analysis

Study participants were requested to remain abstinent for at least two days before contributing a semen sample. The date of semen collection was recorded. 77% of semen samples were collected on the same day as that when the participant contributed the corresponding first urine. 15% were collected 1–6 days prior to urine contribution, 4% were collected 38–51 days prior, and 4% were collected 87–132 days before. Semen samples were collected following masturbation into a sterile plastic specimen cup (NUNC Brand products, USA) and were liquefied at 37°C for 30 min before analysis. Sperm motility, semen volume and sperm concentration were measured according to World Health Organization (WHO) guidelines [28]. Motility parameters were analyzed using a Micro-cell slide and computer-aided semen analysis (CASA, WLJX 9000, Weili New Century Science & Tech Dev., Beijing, China). According to WHO recommendation, percent motile sperm was scored using categories A (rapid progressive motility), B (slow progressive motility), C (nonprogressive motility), and D (immotility). The category C group was excluded in this analysis since it is not an independent variable. In addition, previous studies have shown that the percentage of progressively motile spermatozoa (A + B) is the most significant parameter in relation to the male fertility [29]. Therefore, subsequent analysis was conducted employing two designated groupings for motility, A + B and D. Sperm morphology was determined using the strict criteria described by Kruger and colleagues [30].

### Arsenic (As) determination with HPLC-ICP-MS

Five As species in urine were simultaneously measured by Agilent 1200 Series liquid chromatography (Agilent Technologies, USA) coupled with Agilent 7500 cx inductively coupled plasma mass spectrometry (Agilent Technologies).

The standards (CH_3_)_2_AsOOH, (CH_3_)AsO(OH)_2_, Na_2_HAsO_4_·7H_2_O, As_2_O_3_ and AsB (40 mg/l) were purchased from the National Research Centre for Certified Reference Material in China. Stock solutions were prepared as described. An appropriate amount of (CH_3_)_2_AsOOH, (CH_3_)AsO(OH)_2_ and Na_2_HAsO_4_·7H_2_O were each individually dissolved in deionised water (18.2 MΩ). As_2_O_3_ was dissolved in NaOH (0.1 N) to prepare 1000 mg L^-1^ of DMA^V^, MMA^V^, As*i*^V^ and As*i*^III^. All stock solutions were stored at 4°C in polyethylene bottles. A series of standard As solutions, with a range of 0.1-50 μg L^-1^, were prepared daily by mixing stock solutions.

A pre-column (11.2 mm, 12–20 μm) and a Hamilton PRP-X100 anion-exchange column (250 mm × 4.1 mm, 10 μm) were used to separate AsB, DMA^V^, MMA^V^, As*i*^III^ and As*i*^V^ using a flow rate of 1.0 ml/min. The mobile phase was prepared with 10 mM ammonium nitrate and 10 mM diammonium hydrogen phosphate, which had been adjusted to pH 9.25 by ammonium hydroxide. The mobile phase was never stored for >3 days at 4°C prior to use. The frozen urine samples were defrosted at room temperature for 2 h prior to analysis. The samples were vortexed, and then, 1 ml of urine was filtered through a 0.22 μm polyether sulfone membrane filter and poured into a 1.5 ml autosampler vial [31]. The injection volume was 20 μl. ICP-MS was operated at a generator power of 1500 W. The cooling gas flow, carrier gas flow and makeup gas flow were 15, 1.0 and 1.1 L min^-1^, respectively. The ICP-MS instrument did not run tests in the collision cell mode because the optimal chromatographic parameters employed in this study separated the ^40^Ar^35^Cl peak from the peaks of the other As species [32]. The m/z 75 for As and m/z 115 for the internal standard of indium (In) were monitored with ICP-MS. The peak sequence was listed as AsB, As*i*^III^, DMA, MMA and As*i*^V^, and a peak of ArCl^+^ (m/z = 75) was eluted between MMA and As*i*^V^.

At the start of sample analysis, calibration curves for As concentrations of 0, 0.1, 0.5, 1, 5, 10, 20 and 50 μg L^-1^ were analyzed. The standard reference (0.1 and 1 ppb) was injected into every tenth sample to assess instrumental sensitivity and stability. In addition, the calibration blank was measured to assess any background carryover, and duplicate samples were analyzed to observe reproducibility during analysis. The spiked As species (10 μg L^-1^ in 3 urine samples) had a recovery range of 88-105%. The limit of detection (LOD) for the AsB, MMA, DMA and As*i*^III^ method was 0.2 μg L^-1^; the As*i*^V^ LOD was 0.5 μg L^-1^.

### Data analysis

**Figure 1:**
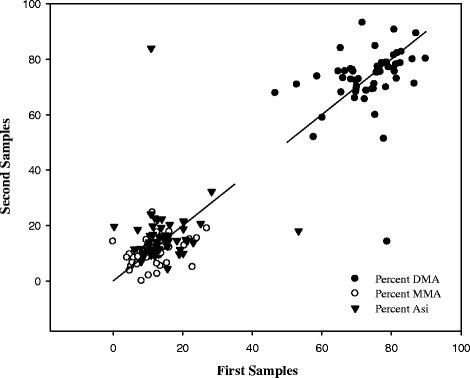
**Correlations of %DMA, %MMA and %As*i*(per cent DMA, per cent MMA and per cent As*i*, respectively) between the repeat samples from study participants.**

In this study, As concentrations adjusted for urine creatinine were used for all statistical analyses. Creatinine levels were determined by a chemoluminescence immunoassay. The normal distribution of the data was verified by the Shapiro-Wilk test. Because of the low detectable frequency for As*i*^V^, total inorganic As (As*i*, the sum of As*i*^III^ and As*i*^V^) was used in the data analysis. The percentage of each As species (%DMA, %MMA, and %As*i*) was calculated by dividing their corresponding concentration by the total As metabolites in urine (the sum of DMA, MMA, and As*i*). The As methylation indices are defined as the primary As methylation index (PMI; MMA/As*i*) and the secondary As methylation index (SMI; DMA/MMA) [33,34]. The averaged measurements of the two urine samples from each participant were previously reported [35], except for the data shown in Figure 1. Continuous variables between two groups (*e.g.*, drinking *versus* non-drinking, smoking *versus* non-smoking) were compared using the Student’s *t*-test. Non-normally distributed variables were analyzed using the nonparametric Mann–Whitney *U*-test. As species concentrations were logarithmically transformed to increase symmetry and normal distribution [36]. The effects of adjusted As species and methylation indices on semen quality with regard to age, smoking/drinking status and abstinence duration were studied using binary logistic regressions, where the continuous variables were dichotomized with cut-offs of the median. For each of the three sperm quality parameters, the data above the reference were used as the controls, without regard to the other two parameters. The statistical package of SPSS 18.0 for Windows was used for all statistical analyses.

## Results

### Demographics and semen diagnosis

The participants’ demographic characteristics and semen parameters are listed in Table 1. All study participants answered the structured questionnaire and completed the semen quality test. Participant ages ranged from 23 to 43 years, with an average age of 32 years. Although it has been suggested that creatinine concentration (0.62±0.53 g L^-1^) correlates with As exposure [37], our findings do not support this assertion. Heavy smokers and drinkers had higher BMIs than other participants. Thirty-two men (33.3%) had normal (above-reference values) semen parameters.

**Table 1:** Subject demographics and distribution of semen parameters (Mean ± SD)

	**Number (%)**	**Mean ± SD**
All	96 (100)	
Age (years)		32 ± 4.8
BMI (kg m^-2^)		23.5 ± 3.27
Creatinine (g L^-1^)		0.98 ± 0.60
Smoking status		
Smoker	38 (40)	
Non-smoker	58 (60)	
Alcohol drinking status		
Drinker	57 (59)	
Non-drinker	39 (41)	
Education status		
>high school	54 (56)	
≤high school	42 (44)	
Semen parameters ^a^		
Sperm concentration (million mL^-1^)^b^		72.3 ± 48.6
Subjects <20 million mL^-1^	13 (14)	
Sperm motility		63.6% ± 19.4%
Subjects <50% motile sperm	24 (25)	
Semen volume (mL)		2.74 ± 0.88
Subjects <2 mL	13 (14)	
Normal sperm morphology rate		16.6% ± 8.8%
Subjects <15%	45(47)	
Abstinence time		
2 days	9 (9)	
3 days	22 (23)	
4 days	15 (16)	
5 days	24 (25)	
≥6 days	26 (27)	

^a^ WHO guidelines for sperm concentration, motility, volume and sperm morphology are sperm concentration ≥20×10^6^/mL, motility ≥50% motile, semen volume ≥2 mL, and Normal sperm morphology rate ≥15%.

^b^ sperm concentration, sperm motility and normal sperm morphology rate are non-normally distributed. The medians are 66.6, 68.8% and 15.2%, and the 95% percentiles are 62.5 ~ 82.2, 59.7% ~ 67.5% and 14.7 ~ 18.4%, respectively.

### Arsenic (As) species concentrations and profile

Table 2 shows the geometric mean concentrations and proportions of As species stratified by groups based on smoking and drinking. Because of the narrow age range, data were not age-adjusted when comparing the differences between the variables. The results of the Student’s *t*-test and the Mann–Whitney *U*-test, which are expressed in *P*-values, are listed below the corresponding variables. The geometric mean concentrations (expressed as μg g^-1^ creatinine) of urinary AsB, DMA, MMA and As*i* for all participants were 7.49, 20.9, 2.77, and 4.03 μg g^-1^, respectively, while the mean concentrations (± SD, standard deviation) were 14.0 (±24.8), 26.2 (±13.7), 3.66 (±3.33) and 4.89 (±3.67) μg g^-1^, respectively. The geometric mean percentages of DMA, MMA and As*i* for all study participants, calculated from non-creatinine adjusted urinary arsenic concentrations were 75.4%, 10.3% and 14.3%, and they were very similar to creatinine-adjusted As concentration values presented in Table 2. There were no significant differences in AsB levels between all pairs of demographic variables. The differences in MMA and %MMA between smokers and non-smokers were significant. Consumption of alcohol had no detectable impact on As species concentrations and profiles in the urine.

**Table 2:** **Differences in As species (geometric means ± SD) by demographic variables**^**ª**^

	**Urinary concentration (μg g**^**-1**^**creatinine)**						
**Variable**	**AsB**	**DMA**	**MMA**	**As*i***	**%DMA**	**%MMA**	**%As*i***
All	7.49±24.8	20.9±13.7	2.77±3.33	4.03±3.67	73.4±8.44	9.73±4.65	14.2±6.88
25th	3.41	14.8	1.76	2.82	70.3	7.51	11.8
50th	6.63	20.7	2.59	3.81	75.4	10.1	14.2
75th	13.0	30.9	4.19	5.79	78.9	12.9	16.6
Smoker	6.91±25.9	18.7±11.9	2.22±3.45	3.61±3.75	73.6±9.88	8.72±4.37	14.2±8.17
Non-smoker	7.94±27.7	22.6±14.8	3.24±3.22	4.36±3.63	73.3±7.33	10.5±4.04	14.2±5.86
*P*-value	0.60	0.12	0.01** ^b^	0.16	0.29	0.04*	0.94
Drinker	7.28±19.7	21.3±12.7	2.78±3.47	3.89±3.66	74.2±7.90	9.67±4.99	13.5±5.73
Non-drinker	7.82±34.8	20.3±15.3	2.76±3.15	4.25±3.74	72.3±9.19	9.82±4.17	15.1±8.21
*P*-value	0.85	0.67	0.96	0.51	0.55	0.83	0.34

^a^ The statistical difference was listed with *P*-value.

Proportion of each As species (%DMA, %MMA, and %As*i*) were calculated by dividing their concentration by the total As metabolites in urine (the sum of DMA, MMA and As*i*).

^b^ The comparison which the *P*-value applies are smoking habit and dinking habit, respectively, **Difference is significant at the 0.01 level; *Difference is significant at the 0.05 level.

The Spearman and log-scale Pearson correlation coefficients between the two As species are listed in Table 3. The maximum correlation coefficient (R) between MMA and As*i* was 0.82. There were strong correlations in DMA, MMA and As*i.* The correlation coefficients for AsB, DMA, MMA and As*i* were significant (*P* <0.01).

**Table 3:** Spearman and log-scale Pearson correlations between different arsenic species

	**Spearman**				**Pearson (log-scale)**			
	**AsB**	**DMA**	**MMA**	**As*i***	**AsB**	**DMA**	**MMA**	**As*i***
AsB	1	0.47**	0.54**	0.48**	1	0.52**	0.46**	0.49**
DMA		1	0.74**	0.70**		1	0.76**	0.73**
MMA			1	0.76**			1	0.82**
As*i*				1				1

** means that at the 0.01 confidence level (two sides), the correlations is significant.

The statistical analysis of the two samples contributed per study participant showed that the As species concentrations did not exhibit a significant correlation, except AsB (R = 0.59, *P* <0.01). When comparing the percentages of the species between the two samples, a stronger correlation for %DMA (R = 0.47, *P* <0.01), %MMA (R = 0.45, *P <*0.01) and %As*i* (R = 0.23, *P* =0.09) was observed when compared with the previously-mentioned species concentrations (Figure 1).

### Methylation index profile

The primary As methylation index (PMI) and the secondary As methylation index (SMI) were mutually compared between the different groups based on demographic variables. Although have there was a significant difference for MMA and %MMA between smoker and the non-smoker categories (Table 2), the difference for PMI was not significant. However, a significant difference for SMI between smokers *versus* non-smokers (*P* =0.05) was observed.

### Arsenic (As) species *versus* semen parameters

Adjusted odds ratios (ORs) for the relationships between dichotomized semen parameters and creatinine-adjusted As species are presented in Table 4. We observed that DMA concentrations above the median were significantly associated with below-reference sperm concentration (ORs: 1.0 - 7.2; *P* =0.02, corrected for age, BMI, abstinence, smoking and drinking) as well as DMA% and sperm concentration (ORs: 1.0 - 7.0; *P* =0.02).

**Table 4:** **Association of the below-reference values**^**ª**^**of sperm concentration, sperm motility and semen volume with dichotomised of As levels (n = 96)**^b^

	**Sperm Concentration (n = 13)**		**Sperm Motility (n = 24)**		**Semen Volume (n = 13)**	
	**No.**	**Adjusted OR (95% CI)**	**No.**	**Adjusted OR (95% CI)**	**No.**	**Adjusted OR (95% CI)**
AsB						
1	10	1.0	11	1.0	10	1.0
2	3	0.3 (0.07-1.5)	13	1.4 (0.5-4.0)	3	2.0 (0.5-9.0)
*P*-value		0.16		0.48		0.34
DMA						
1	3	1.0	12	1.0	7	1.0
2	10	7.2 (1.4-37.1)	12	1.1 (0.4-2.8)	6	0.9 (0.3-3.1)
*P*-value		0.02		0.89		0.89
MMA						
1	9	1.0	13	1.0	8	1.0
2	4	0.3 (0.07-1.3)	10	0.8 (0.3-2.2)	5	0.7 (0.2-2.5)
*P*-value		0.10		0.66		0.58
As*i*						
1	8	1.0	12	1.0	6	1.0
2	5	0.6 (0.1-2.2)	12	1.1 (0.4-2.8)	7	1.4 (0.4-4.8)
*P*-value		0.41		0.87		0.59
%DMA						
1	3	1.0	14	1.0	6	1.0
2	10	7.0 (1.4-34.6)	10	0.6 (0.2-1.5)	7	1.1 (0.3-3.6)
*P*-value		0.02		0.28		0.92
%MMA						
1	7	1.0	10	1.0	8	1.0
2	6	0.6 (0.2-2.6)	14	1.7 (0.7-4.6)	5	0.6 (0.2-2.3)
*P*-value		0.51		0.27		0.50
%As*i*						
1	9	1.0	10	1.0	5	1.0
2	4	0.3 (0.08-1.3)	14	1.7 (0.6-4.3)	8	1.8 (0.5-6.3)
*P*-value		0.12		0.29		0.35
PMI						
1	7	1.0	11	1.0	8	1.0
2	7	0.5 (0.1-2.3)	13	1.2 (0.5-3.3)	5	0.7 (0.2-2.3)
*p*		0.40		0.68		0.51
SMI						
1	5	1.0	14	1.0	5	1.0
2	8	3.0 (0.7-13.5)	10	0.6 (0.2-1.6)	8	3.0 (0.7-13.5)
*P*-value		0.15		0.31		0.15

^a^ Comparison group is at or above reference value for sperm concentration (≥20×10^6^/mL), motility (≥50% motile), and semen volume (≥2 mL).

^b^ Adjusted for age (continuous), BMI (continuous), abstinence time (5 categories: 2, 3, 4, 5, and ≥6 days), smoking habit (non-smoker and smoker), drinking habit (non-drinker and drinker).

## Discussion and Conclusions

Urinary As species have been used as indicators of human exposure to As [38]. Analyzing the urinary profile of AsB, DMA, MMA, As*i*^III^ and As*i*^V^ could yield information on the exposure sources and dietary origin of these inorganic and organic species [31,39]. The profile could also lend important insights into understanding As metabolism and its effects on human health [40]. The urinary species profile of %DMA, %MMA and %As*i* are observed to be fairly stable over time and are useful in understanding the relationship between As exposure and health consequences [41]. Some researchers have identified associations between As’ methylating capacity (urinary %MMA) and related cancer risks [3,42,43].

### Source analysis of exposure to arsenic (As)

Urinary As*i*^III^ is usually associated with the consumption of mineral water, and urinary AsB is usually associated with the consumption of seafood. In this study, As*i*^III^ and As*i*^V^ concentrations were lower than the published data for populations exposed to As in As*i*-contaminated drinking water [44]. The average concentration of total As in drinking water was 0.023 mg L^-1^ in Chongqing [45]. Because our participants were not exposed to an As-polluted geological environment, dietary intake could be the main source of exposure. The urinary concentrations of AsB and DMA (geometric mean AsB 7.49 and median DMA 20.9 μg L^-1^) in our cohort were lower than that reported for individuals who had a preference for seafood intake (median AsB 61.3 and 74.5, median DMA 41.1 and 42.6 μg L^-1^, respectively) [32,46]. The mutually significant correlation of all urinary As species (Table 3) suggests a common source of exposure [39], and the significant correlations of AsB and As*i*^III^ between the two contributed samples support a reasonably stable route of exposure. Previous studies have shown that rice can effectively concentrate As from soil [47]; the total As concentrations observed in polished (white) rice from Sichuan province (Chongqing previously belonged to Sichuan province) ranged from 0.043 to 0.208 μg g^-1^, and the inorganic As concentration ranged from 0.034 to 0.167 μg g^-1^[48]. With a rice consumption of 0.5 kg day^-1^ and a water consumption of 2 L day^-1^ for adults with an average body weight of 65 kg, the daily intake of inorganic As was about 0.26-1.28 μg kg^-1^. This dose exceeded the daily oral As reference dose of 0.3 μg kg^-1^[49]. Because rice is the staple food for our participants, it is possible that their primary inorganic As source derives from this exposure route. For AsB, the subjects may be exposed to AsB from dry brown algae and agar-agar because fresh seafood is typically unavailable for inland inhabitants. However, the human body does not metabolize it, but rather excretes it from urine directly [50,51].

### Species metabolism and methylation indices

The correlations among As*i*, MMA and DMA are stronger than the correlations between AsB and As*i*, MMA and DMA (Table 3), which suggests that the study participants metabolized As*i* into MMA and DMA. Between smokers and non-smokers, there are significant differences in MMA concentrations and %MMA, as smokers exhibit lower MMA concentrations. This difference disappeared when the difference of MMA is adjusted by As*i* (*i.e.*, PMI), but when it is adjusted by DMA (*i.e.*, SMI) the difference is still significant as smokers have a higher SMI. This observation supports the notion that smoking may increase levels of As metabolites [31,52]. However, we didn’t observe an association between As*i* and smoking [53,54], which may suggest that the urinary As concentration was a consequence of arsenical pesticide used on tobacco. Drinking shows no effect on the As species’ concentrations and profiles in the participants. This result is in accordance with previous studies [42]. These findings suggest that As exposure from alcohol may be negligible.

### Arsenic (As) species associated to semen parameters

It has been suggested that As may exert an influence on the endocrine system. Arsenite can inhibit the growth of MCF-7 cells by reducing the growth stimulatory effects of oestrogen [15]. Sodium arsenite treatment also decreases circulating levels of oestradiol in rats [16]. Furthermore, the steroidogenic dysfunction after As exposure has been reported to result in infertility in male rats [21-24]. This may suggest a similar effect in humans. However, few population studies have been reported examining this possibility [25].

Among the investigated associations between the information on As burden [species (AsB, DMA, MMA, As*i*), species percentages (%DMA, %MMA, %As*i*) and methylation indices (PMI and SMI)] and semen parameters (sperm concentration, sperm motility and semen volume), the dosage correlations between DMA or %DMA *versus* sperm concentrations are similarly significant. The higher DMA concentrations may correlate with lower sperm concentrations. An *in vitro* experiment indicated that organic arsenicals exhibited rapid sperm immobilizing activity [55]. The As-treated animals exhibited decreased sperm counts, sperm motility and testicular weight when compared with untreated animals [56]. As disrupts the process of meiosis and post-meiotic stages of spermatogenesis, and acute exposure causes rapid and extensive disruption of spermatogenesis in mice [24]. Our results suggest that exposure to environmental levels of arsenic may also result in low sperm concentrations in humans. However, there are no associations between DMA concentrations and sperm motility or semen volume. As one of the main As species, DMA is the metabolic product of As in humans. It is unclear whether it is DMA itself or the process of As metabolism that affects sperm production. Recently, rodent model experiments have indicated that As impairs male reproductive functions by inducing oxidative stress [57], but the mechanism in human has not yet been verified.

For the first time reported, we demonstrate that increasing exposure to the As species DMA inversely correlates with sperm levels in humans. The study participants were recruited from hospital patients and not from the general population, which may introduce bias; however, these are otherwise healthy individuals. The correlations of As concentrations between the first and second contributed urine samples were significant although weakly so, which was unexpected. That may be attributed to different times of day for sample collection, an occasional higher exposure dose from something such as seafood, or the relatively small sample size. The result may also be inflated by the multiple comparisons. However, moderate associations between As exposure and semen quality may have been overlooked because of the small sample size. Due to the ubiquitous exposure to As, we recommend that these initial results should be confirmed in the larger population study.

## Conclusions

As is a well-known carcinogen that has been recognized as an endocrine disrupter at environmental levels. In China and throughout Asia, huge populations depend on rice as a staple food, which can concentrate higher levels of inorganic As in the grain than wheat. Rice may be the main source of As intake in these subjects. For the first time, we found that the general As exposure may be associated with reduced human semen quality. By linking these cases, we can see that the general population may be exposed to inorganic As *via* rice, and its endocrine disrupting effects may further decrease semen quality. Because of the spread of As in the environment, further research is urgently needed to fully understand its health effects on the semen quality.

## **Abbreviations**

AsB, Arsenobetaine; DMA, (CH3)2AsOOH; MMA, (CH3)AsO(OH)2; Asi, Inorganic arsenic; PMI, The primary arsenic methylation index (MMA/Asi); SMI, The secondary arsenic methylation index (DMA/MMA); ORs, Odds ratios.

## Misc

Weipan Xu and Huaqiong Bao contributed equally to this work.

## Competing interests

The authors declare that they have no competing interest.

## **Authors’ contributions**

WX and FL carried out the As determination, performed the statistical analysis and drafted the manuscript. HB, MC, LL and CL participated in the subject recruitment, urine sample collection, questionnaire and semen analysis. LL carried out the determination of urine creatinine and performed the statistical analysis. HS conceived the study, and participated in the design and coordination of the study, and helped to draft the manuscript. YGZ participated in the study design. JS and SD helped to draft the manuscript. All authors read and approved the final manuscript
